# Linear Mixed-Effect Models Through the Lens of Hardy–Weinberg Disequilibrium

**DOI:** 10.3389/fgene.2022.856872

**Published:** 2022-04-12

**Authors:** Lin Zhang, Lei Sun

**Affiliations:** ^1^ Department of Statistical Sciences, University of Toronto, Toronto, ON, Canada; ^2^ Division of Biostatistics, Dalla Lana School of Public Health, University of Toronto, Toronto, ON, Canada

**Keywords:** genome-wide association study, dependent sample, robust association analysis, heritability estimate, Hardy–Weinberg equilibrium

## Abstract

For genetic association studies with related individuals, the linear mixed-effect model is the most commonly used method. In this report, we show that contrary to the popular belief, this standard method can be sensitive to departure from Hardy–Weinberg equilibrium (i.e., Hardy–Weinberg disequilibrium) at the causal SNPs in two ways. First, when the trait heritability is treated as a nuisance parameter, although the association test has correct type I error control, the resulting heritability estimate can be biased, often upward, in the presence of Hardy–Weinberg disequilibrium. Second, if the true heritability is used in the linear mixed-effect model, then the corresponding association test can be biased in the presence of Hardy–Weinberg disequilibrium. We provide some analytical insights along with supporting empirical results from simulation and application studies.

## 1 Introduction

Genetic association tests are often derived from a regression model, regressing the phenotypic data of a complex trait (*Y*) on the genotypic data of a single-nucleotide polymorphism (SNP; *G*), as well as on the covariate data of important environmental factors (*Z*). When individuals in a sample are genetically related with each other, the linear mixed-effect model (LMM) is the most commonly used method for genome-wide association studies (GWAS) (Eu-Ahsunthornwattana et al., 2014). The variance–covariance matrix of the regression model is partitioned into a weighted sum of the genetic correlation matrix and the correlation matrix due to shared environmental effects. The genetic correlation matrix is typically represented by the kinship coefficient matrix, which is either inferred from the (correctly) known pedigree structure or estimated based on the available genome-wide genetic data (Yang et al., 2011; Dimitromanolakis et al., 2019). The weight for the genetic correlation matrix is referred to as the heritability of the trait (Visscher et al., 2006; Visscher et al., 2008); Falconer (1985) gave a theoretical modeling of the variance partition, which sets the foundation for heritability.

It is commonly assumed that these regression-based association tests are robust to departure from Hardy–Weinberg equilibrium (HWE) (Sasieni, 1997). HWE states that the two alleles in a genotype are independent draws from the same Bernoulli distribution, or, equivalently, genotype frequencies depend solely on the allele frequencies (Hardy et al., 1908; Weinberg, 1908). For a biallelic SNP with two possible alleles *A* and *a*, let *p* and 1 − *p* be the population allele frequencies, respectively. Under HWE, *p*_*aa*_ = (1 − *p*)^2^, *p*_*Aa*_ = 2*p*(1 − *p*), and *p*_*AA*_ = *p* ^2^, where *p*_*aa*_, *p*_*Aa*_, and *p*_*AA*_ are the population genotype frequencies of genotypes *aa*, *Aa*, and *AA*, respectively. To quantify the departure from HWE or the amount of Hardy–Weinberg disequilibrium (HWD),

$$\delta =p_{AA}-p^{2}$$ (1)

is a widely used measure (Weir, 1996), and *δ* = 0 indicates HWE holds. We note that a) HWE is also known as Hardy–Weinberg proportion and b) *δ* is also known as *p*(1 − *p*)*F*, where *F* is the inbreeding coefficient (Powell et al., 2010). Equivalently, instead of quantifying the genotype frequencies as *p*_*aa*_ = (1 − *p*)^2^ + *δ*, *p*_*Aa*_ = 2*p*(1 − *p*) − 2*δ*, and *p*_*AA*_ = *p* ^2^ + *δ* based on *δ* (Weir, 1996), we can define them based on *F* as *p*_*aa*_ = (1 − *p*)^2^ + *p*(1 − *p*)*F*, *p*_*Aa*_ = 2*p*(1 − *p*)(1 − *F*), and *p*_*AA*_ = *p* ^2^ + *p*(1 − *p*)*F* (Powell et al., 2010). As the classical Pearson *χ* ^2^ HWE testing is based on comparing the observed genotype counts with the expected under HWE (Zhang and Sun, 2021); we thus chose *δ* for this work to be consistent with the GWAS literature.

A truly associated or causal SNP can be out of HWE (Wittke-Thompson et al., 2005; Ryckman and Williams, 2008; Turner et al., 2011), which is often overlooked but an important consideration when studying a method’s robustness to HWD. Note that the HWD attributed to true association is typically not as extreme as the HWD caused by genotyping errors (Zhang and Sun, 2020). Thus, true HWD can remain in a “cleaned” dataset after applying the standard HWD-based quality control screening using a stringent *p*-value threshold [e.g., 10^–12^ for an application of the UK Biobank data by Bycroft et al. (2018)]. With a sample of independent individuals, both theoretical and empirical results support that genotype-based association tests are robust to HWD (Sasieni, 1997; Schaid and Jacobsen, 1999; Zhang, 2021). However, in the presence of sample dependency, little has been discussed.

In this report, we first provide some analytical insights on why the standard LMM can be sensitive to HWD in pedigree data in contrast to when analyzing a sample of unrelated individuals. We then demonstrate with a simple sib-pair design that 1) when the heritability is estimated from the data as in practice, although the empirical type I error rate of the LMM is well controlled, the estimated heritability is biased, often upward biased; 2) when the true heritability is known and used, the empirical type I error rate of the LMM is then inflated when *δ* > 0, and deflated if *δ* < 0. The result of 2) is novel, but it is mostly of an academic interest as the true heritability of a trait is often unknown in practice. On the other hand, the result of 1) has important practical implications because if the estimate of a trait heritability is larger than the true value, then it helps explain some of the “missing heritability” (Manolio et al., 2009); the insightful work of Chen (2014) “discuss[es] the circumstances in which the HE [Haseman‐Elston] regression and the mixed linear model are equivalent.”

## 2 Methods

### 2.1 Traditional *Y* ∼ *G* Model With Independent Samples, *T*_Indep_, Is Robust to HWD

Let *Y* be a (continuous) trait of interest, and *G* = 0, 1, and 2, respectively, for the genotypes *aa*, *Aa*, and *AA* of a SNP. Additionally, for notation simplicity but without loss of generality, we assume that there is only one additional covariate, denoted by *Z*. With a sample of *n* unrelated individuals, the traditional genotype-based association analysis assumes that

$$y=\alpha^{*}1+\beta^{*}\text{g}+\gamma^{*}\text{z}+\epsilon^{*},\;\epsilon^{*}∼N\left(\text{0},\sigma^{*2}I\right),$$ (2)

where y = (*y*_1_, *y*_2_,…, *y*_*n*_) is a *n* × 1 vector for the phenotypic values, 1 is a *n* × 1 vector of 1’s, g = (*g*_1_, *g*_2_,…, *g*_*n*_) is a *n* × 1 vector for the genotypes of the SNP, z = (*z*_1_, *z*_2_,…, *z*_*n*_) is a *n* × 1 vector for the covariate values, ϵ* is the error term with variance σ*^2^, and *I* is the identity matrix.

Score-based tests are often used for genetic association analyses (Derkach et al., 2015). In this case, the score statistic of testing *H*_0_: β* = 0 can be easily derived as

$$T_{\text{ indep}}=n\cdot \frac{{\left\{{\left(g-\bar{g}1\right)}^{T}\left(\text{y}-\bar{y}1\right)-\frac{{\left(g-\bar{g}1\right)}^{T}\left(\text{z}-\bar{z}1\right){\left(\text{y}-\bar{y}1\right)}^{T}\left(\text{z}-\bar{z}1\right)}{{\left(\text{z}-\bar{z}1\right)}^{T}\left(\text{z}-\bar{z}1\right)}\right\}}^{2}}{\left[{\left(g-\bar{g}1\right)}^{T}\left(g-\bar{g}1\right)-\frac{{\left\{{\left(g-\bar{g}1\right)}^{T}\left(\text{z}-\bar{z}1\right)\right\}}^{2}}{{\left(\text{z}-\bar{z}1\right)}^{T}\left(\text{z}-\bar{z}1\right)}\right]\left[{\left(\text{y}-\bar{y}1\right)}^{T}\left(\text{y}-\bar{y}1\right)-\frac{{\left\{{\left(\text{y}-\bar{y}1\right)}^{T}\left(\text{z}-\bar{z}1\right)\right\}}^{2}}{{\left(\text{z}-\bar{z}1\right)}^{T}\left(\text{z}-\bar{z}1\right)}\right]}.$$ (3)

To observe *T*_indep_’s connection with Hardy–Weinberg disequilibrium, it is instructive to employ some algebraic tricks and show that

$$\frac{1}{n}{\left(g-\bar{g}1\right)}^{T}\left(g-\bar{g}1\right)=\hat{\text{var}}\left(G\right)=2\left(\hat{p}\left(1-\hat{p}\right)+\left({\hat{p}}_{AA}-{\hat{p}}^{2}\right)\right)=2\left(\hat{p}\left(1-\hat{p}\right)+\hat{\delta }\right).$$

Because $\hat{\delta }={\hat{p}}_{AA}-{\hat{p}}^{2}$ measures the amount of HWD present in the data (Weir, 1996), *T*_indep_ inherently adjusts for departure from HWE through $\hat{\text{var}}\left(G\right)=2\left(\hat{p}\left(1-\hat{p}\right)+\hat{\delta }\right)$ . As a result, the traditional genotype-based association test is robust to HWD in independent samples.

When *Y* is binary, the classic logistic regression is commonly used. However, Chen (1983) showed that under some regularity conditions, the score test statistics have an identical form for the exponential family in independent samples, which was recently validated by Zhang and Sun (2021) for genetic association studies. Additionally, Derkach et al. (2015) showed that for *Y*-dependent sampling, “the score statistics are identical for conditional and full likelihood approaches, and are of the same form as those for ordinary random sampling.” Thus, in terms of association testing (not genetic effect estimation), we can conclude that genotype-based association studies of binary traits in independent samples are also robust to HWD.

### 2.2 Linear Mixed-Effect Model With Dependent Samples, *T*_LMM_, Can Be Sensitive to HWD

Although a pedigree-based study design is rare for genome-wide association studies, individuals can be (cryptically) related with each other even in population-based GWAS (Sun et al., 2017). Omitting related individuals simplifies the association analysis but reduces the sample size and thus power. Instead, Σ_Φ_, the kinship coefficient matrix, can be estimated using the available genome-wide data to capture the sample relatedness between the *n* individuals (Visscher et al., 2006; Yang et al., 2011). The association analysis using the full sample can be conducted using the linear mixed-effect model.

$$\text{y}=\alpha^{*}1+\beta^{*}\text{g}+\gamma^{*}\text{z}+\epsilon^{*},\text{ where }\epsilon^{*}∼N\left(\text{0},\sigma_{y}^{2}\Sigma_{y}\right)\;\;\text{and}\;\;\Sigma_{y}=h^{2}\Sigma_{\Phi }+\left(1-h^{2}\right)I.$$ (4)

Compared with the linear model used for independent samples, var(ϵ*) = σ*^2^ *I* in Eq. 2 is replaced by $\sigma_{y}^{2}\Sigma_{y}$ to reflect the sample dependence. The matrix Σ_*y*_ is a weighted average of two components, where Σ_Φ_ reflects the sample relatedness; naturally, the model is reduced to the linear model of Eq. 2 for independent samples when Σ_Φ_ = *I*. The weight *h* ^2^ is interpreted as the heritability of the trait (Visscher et al., 2008), $h^{2}\sigma_{y}^{2}$ as the phenotypic variation due to (additive) genetic variation, and $\left(1-h^{2}\right)\sigma_{y}^{2}=\sigma_{e}^{2}$ as the phenotypic variation due to environmental variation. The matrix Σ_Φ_ is the kinship matrix, where Σ_Φ_(*i*, *j*) = 2*ϕ*_*i*,*j*_ and *ϕ*_*i*,*j*_ is the kinship coefficient between the *i*th and *j*th samples.

By convention, *h* ^2^ is defined as

$$h^{2}=\frac{\sum \overset{2}{\underset{k}{\beta }}\text{var}\left(G_{k}\right)}{\text{var}\left(Y\right)}=\frac{\sum \overset{2}{\underset{k}{\beta }}2p_{k}\left(1-p_{k}\right)}{\sum \overset{2}{\underset{k}{\beta }}2p_{k}\left(1-p_{k}\right)+\sigma_{e}^{2}},$$

where there could be multiple causal SNPs, *k* = 1,…, *S*. In reality, *h* ^2^ is estimated by the correlation between phenotypes of related individuals. Consider the simple case of sibling pairs, and let *Y*_1_ and *Y*_2_ be the phenotypes for sib 1 and sib 2, respectively. Allowing for HWD and adjusting for the kinship coefficient *ϕ*, the estimated *h* ^2^ is

$${\hat{h}}^{2}=\hat{\text{corr}}\left(Y_{1},Y_{2}\right)/2\phi ,$$

where corr(*Y*_1_, *Y*_2_) depends on the correlation between *G*_1*k*_ and *G*_2*k*_ between the siblings; see Zhang and Sun (2021) for the derivation of corr(*G*_1*k*_, *G*_2*k*_) accounting for kinship coefficient and HWD. Thus,

$$\frac{E\left({\hat{h}}^{2}\right)}{h^{2}}=\frac{\sum \overset{2}{\underset{k}{\beta }}2\left(p_{k}\left(1-p_{k}\right)+\delta_{k}\right)}{\sum \overset{2}{\underset{k}{\beta }}2p_{k}\left(1-p_{k}\right)},$$

and the bias of the *h* ^2^ estimate is

$$E\left({\hat{h}}^{2}\right)-h^{2}=h^{2}\cdot \frac{\sum \overset{2}{\underset{k}{\beta }}\delta_{k}}{\sum \overset{2}{\underset{k}{\beta }}p_{k}\left(1-p_{k}\right)}.$$ (5)

Under the simple case of one causal SNP, the bias is simplified to *h* ^2^ ⋅ *δ*/(*p*(1 − *p*)).

Given the analytical insights provided so far, we then briefly examine the empirical properties of *T*_LMM_ through both application and simulation studies.

## 3 Results

### 3.1 Cystic Fibrosis Sib-Pair Data Application: *T*_LMM_ Has Correct Type I Error but *h* ^2^ Appears to Be Overestimated

We extracted 65 sibling pairs from a cystic fibrosis (CF) gene modifier study (Wright et al., 2011; Sun et al., 2012). The phenotype *Y* of interest is the lung function measurements of the 130 related individuals with CF. In total, there were 570,539 SNPs genotyped using the Illumina 610-Quad Beadchip after applying the standard quality control, including minor allele frequency (MAF) greater than 2%. To stabilize the variance estimation, we additionally required SNPs to have MAF greater than 5%. We then applied *T*_LMM_ to the remaining 505,172 SNPs. In the application, we treated *h* ^2^ as unknown and estimated it based on the linear mixed-effect model of Eq. 4 as in convention.

When *h* ^2^ was estimated from the data, our association testing based on *T*_LMM_ had good type I error control (results not shown), consistent with the empirical observations in the GWAS literature. However, the estimated *h* ^2^, obtained using the 65-pair sibling data, is ${\hat{h}}^{2}=0.82$ . This value is substantially greater than 0.5, the commonly believed “true” heritability of lung function in CF obtained from the classic monozygous (MZ) vs. dizygous (DZ) twin-based estimation method (Vanscoy et al., 2007).

To verify if the large heritability estimate from the LMM method in our application was due to chance, we conducted a proof-of-principle simulation study. We assumed that only one causal SNP, *G*_causal_ with MAF of 0.2, affects *Y* with *h* ^2^ = 0.5. Genotype and phenotype values for 65 sibling pairs were then simulated under the assumption of HWE (i.e., without HWD). Among the 100,000 independently simulated replicates, only 4.24*%* of the heritability estimates were greater than ${\hat{h}}^{2}=0.82$ . This suggests that ${\hat{h}}^{2}=0.82$ , the value that was observed in the CF data application, was unlikely if the true heritability was 0.5 and without HWD at the causal SNP.

To verify if HWD at the causal SNP can lead to a biased heritability estimate, we then conducted additional simulation studies, following the same sib-pair design as mentioned previously. Our goal is to demonstrate that 1) when *h* ^2^ is treated as a nuisance parameter, its estimate based on model (Eq. 4) cross-reference can be biased in the presence of HWD; and 2) assuming the true *h* ^2^ is known, the empirical type I error rate of LMM (Eq. 4) cross-reference inflates when *δ* > 0 and deflates when *δ* < 0.

### 3.2 Simulated Sib-Pair Data in the Presence of HWD: *h* ^2^ Estimate Is Biased

Consider a continuous trait *Y* with *h* ^2^ = 0.5 and influenced by one causal SNP, *G*_causal_, with minor allele frequency of 0.2 and with HWD factor, *δ*_causal_, ranging from −0.04 to 0.16. A non-associated SNP, *G*_tested_, also has an MAF of 0.2 but with its own *δ*_tested_, which may not be the same as *δ*_causal_ in a specific simulation study. The sample size was 65 sibling pairs, chosen to match with the sample size of the cystic fibrosis application study in Section 3.1.

Most practical implementations of the linear mixed-effect model (Eq. 4) cross-reference treat *h* ^2^ as a nuisance parameter, and no type I error issue has been reported. Indeed, when *h* ^2^ was estimated in our simulation study conducted in Section 3.3, the test size of *T*_LMM_ was correct at the nominal level (black squares in Figure 3 shown in Section 3.3) even if *δ*_tested_ ≠ 0 (i.e., out of HWE) and across the range of *δ*_causal_ values (from −0.04 to 0.16).

**FIGURE 1:**
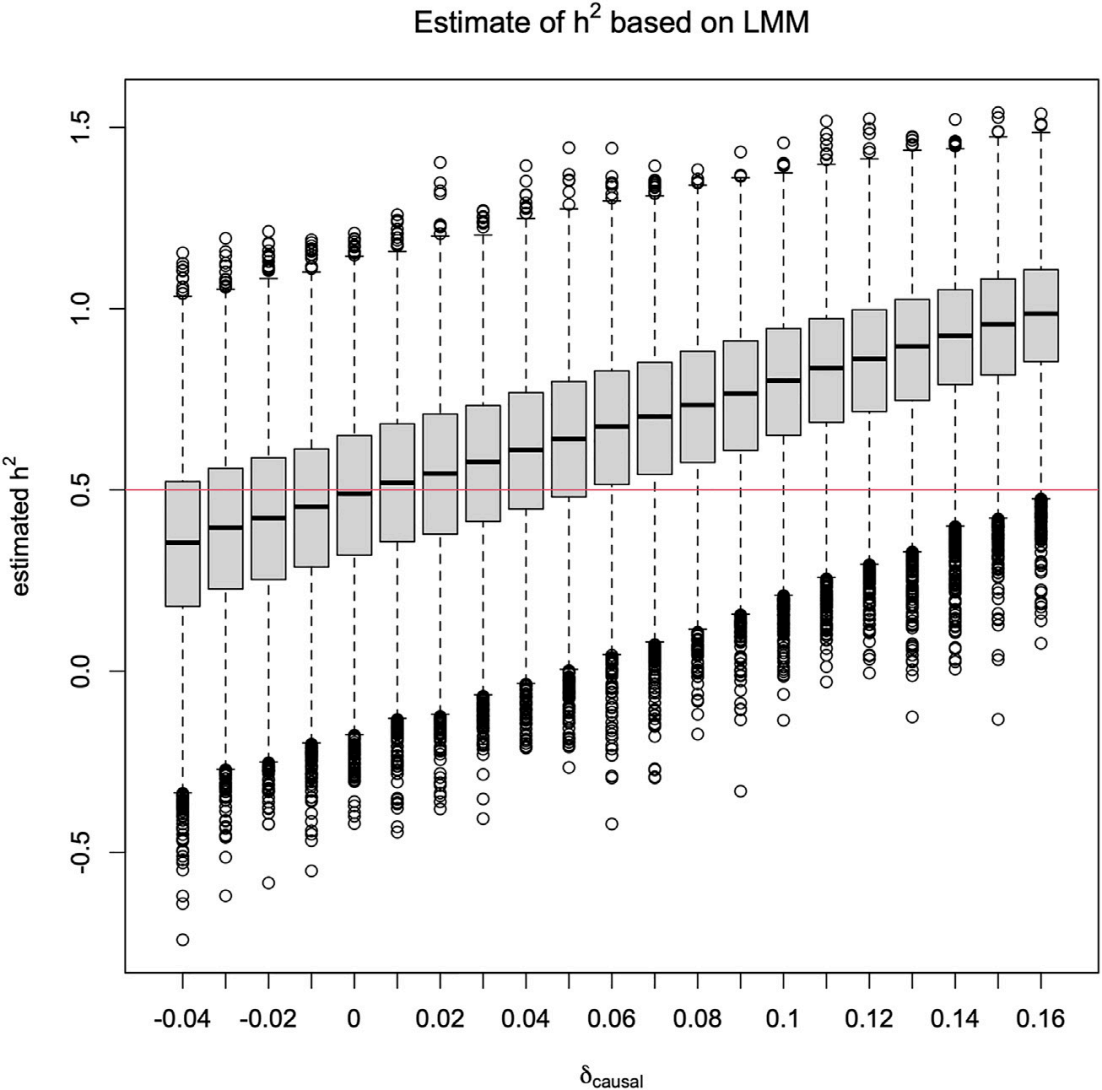
Box plots of ${\hat{h}}^{2}$ , estimated from the linear mixed-effect model (Eq. 4) against ***δ***_**causal**_. The true heritability of the phenotype is *h* ^2^ = 0.5. The minor allele frequencies *p*_causal_ = *p*_tested_ = 0.2 and 10,000 independent replicates of phenotypes and genotypes for 65 sibling pairs were simulated for each *δ*_causal_ value. The empirical type 1 error rates are shown in Figure 3 as black squares.

However, in this situation, when *h* ^2^ is treated as unknown, the impact of HWD is on the estimation of *h* ^2^. Specifically, Figure 1 shows that ${\hat{h}}^{2}$ is downward biased when *δ*_causal_ < 0, and upward biased if *δ*_causal_ > 0. The bias can be substantial. For example, when *δ*_causal_ = 0.10, the estimated heritability ${\hat{h}}^{2}$ is centered at 0.80 as compared to the true value of 0.5, with a bias of 0.30. Indeed, based on our theoretical insight in Section 2.2, the expected bias is *h* ^2^ ⋅ *δ*/(*p*(1 − *p*)) = 0.5 ⋅ 0.1/(0.2(1–0.2)) = 0.31.

In Figure 1, it is notable that ${\hat{h}}^{2}$ can be greater than one. Since *h* ^2^ is the proportion of variance in *Y* explained by additive genetic variation, 0 ≤ *h* ^2^ ≤ 1 by definition. However, if *δ*_causal_ ≠ 0, ${\hat{h}}^{2}$ based on the LMM, without additional truncation, is a biased estimate of *h* ^2^ with a bias of *h* ^2^ ⋅ *δ*/(*p*(1 − *p*)) for this sib-pair design as shown in Section 2.2; the bias is 0 (i.e., no bias) under HWE when *δ* = 0.

**FIGURE 2:**
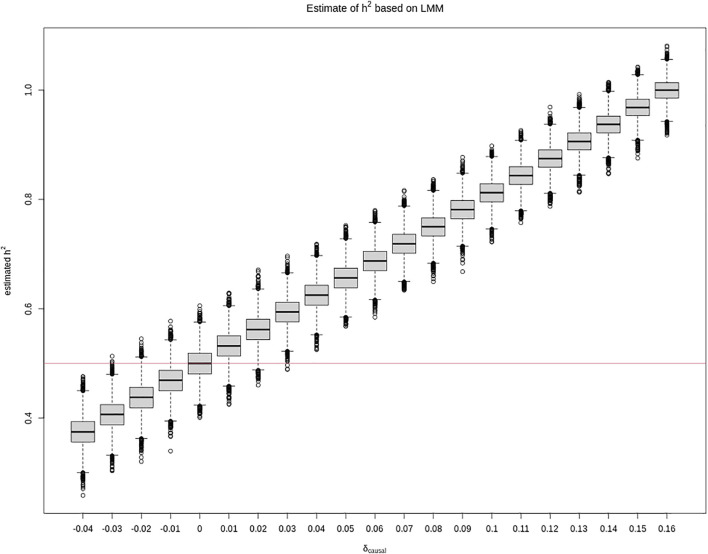
Box plots of ${\hat{h}}^{2}$ , estimated from the linear mixed-effect model (Eq. 4) against *δ*_causal_ using 5,000 sibling pairs. The other parameter values are the same as in Figure 1, where the true heritability of the phenotype is *h* ^2^ = 0.5, the minor allele frequencies *p*_causal_ = *p*_tested_ = 0.2, and 10,000 independent replicates were simulated for each *δ*_causal_ value.

Additionally, although a larger sample that consists of 5,000 sibling pairs shrinks the variance of the *h* ^2^ estimate as expected, it does not shrink the bias, as shown in Figure 2. However, we also note that, in practice, it is unlikely to have so many sibling pairs.

### 3.3 Simulated Sib-Pair Data in the Presence of HWD: When Using the True *h* ^2^ Value *T*_LMM_ Has Incorrect Test Size

Here, we conducted the association analysis between *Y* and the non-associated SNP, *G*_tested_, using the LMM model of Eq. 4 but assuming *h* ^2^ = 0.5 is known.

**FIGURE 3:**
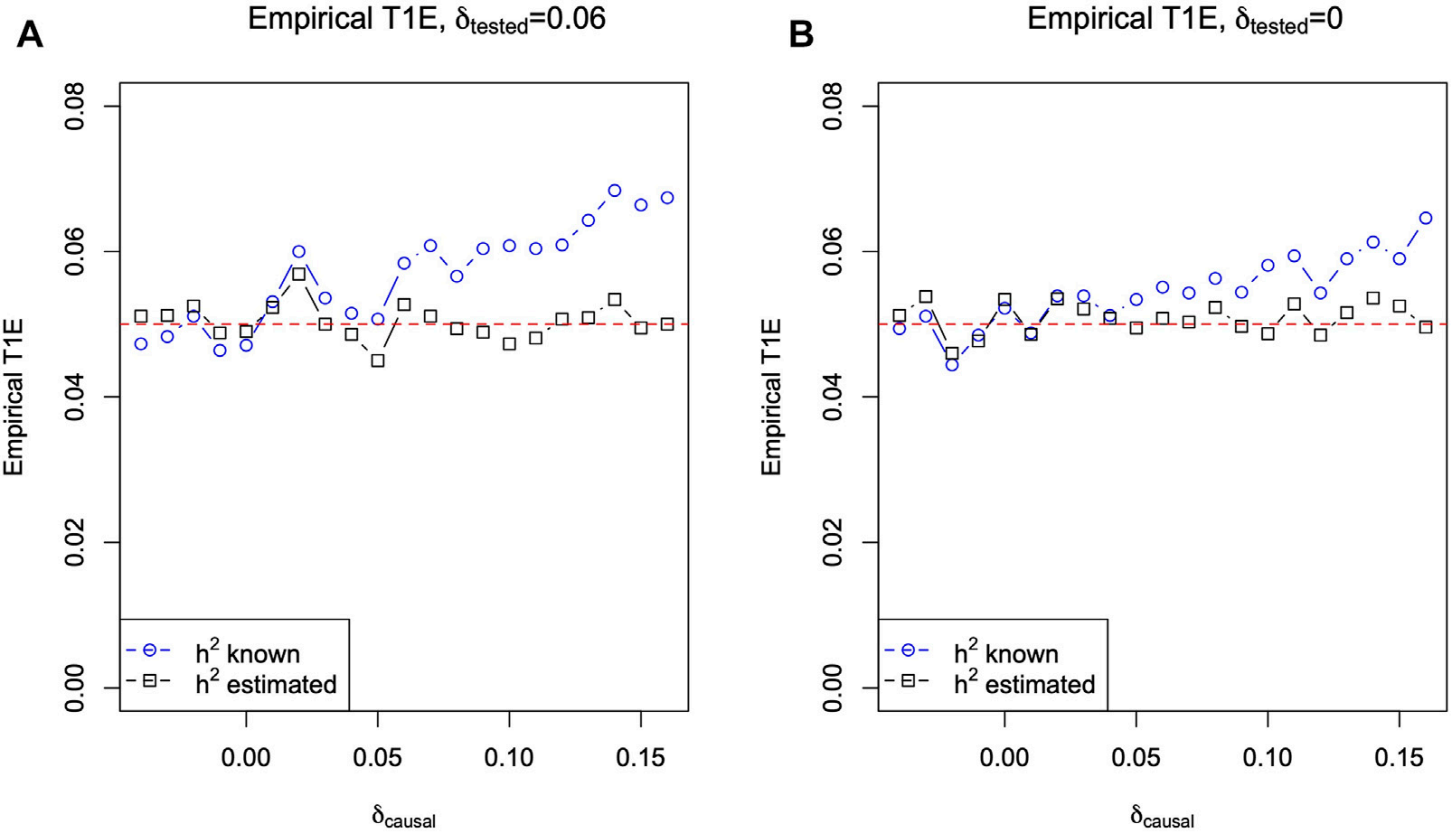
Empirical type I error rate of *T*_LMM_ based on the linear mixed-effect model (Eq. 4) against ***δ***_**causal**_. **(A)** When *G*_tested_ of tested SNPs is in HWD with *δ*_tested_ = 0.06. **(B)** When *G*_tested_ of tested SNPs is in HWE with *δ*_tested_ = 0. The true heritability of the phenotype is *h* ^2^ = 0.5, the minor allele frequencies *p*_causal_ = *p*_tested_ = 0.2, and 10,000 independent replicates of phenotypes and genotypes for 65 sibling pairs were simulated for each *δ*_causal_ value. The blue circles are for *T*_LMM_ using the true heritability *h* ^2^ = 0.5, and the black squares are for *T*_LMM_ while estimating *h* ^2^ (results of ${\hat{h}}^{2}$ are shown in Figure 1).

Figure 3A plots the empirical type I error rates (blue circles) of *T*_LMM_ using the true *h* ^2^ = 0.5, for a nominal level of 0.05, estimated from independently simulated 10,000 replicates for each *δ*_causal_ value. (An empirical type I error greater than 0.05 + 3 ⋅ 0.002 = 0.056 can be considered inflated as the standard error of the empirical type I error rate can be estimated as $\sqrt{0.05\cdot 0.95/10000}=0.002$ .) In Figure 3A, the trend of type I error inflation is clear as *δ*_causal_ increases.

In Figure 3A, we set *δ*_tested_ = 0.06, but we note that the main cause of the type I error issue is *δ*_causal_ ≠ 0 when using the LMM of Eq. 4 with *h* ^2^ = 0.5 plugged in. Indeed, Figure 3B shows that even if *G*_tested_ is in HWE (i.e., *δ*_tested_ = 0), the problem remains, albeit less severe, as long as *δ*_causal_ ≠ 0.

## 4 Discussion

We used a sib-pair design to demonstrate that the linear mixed-effect model can be problematic in the presence of Hardy–Weinberg disequilibrium at the causal SNP(s). To demonstrate that the LMM-based heritability estimate can be biased, as a proof-of-principle, our simulation study assumed that the phenotype *Y* has only one causal SNP, which is unrealistic for complex traits. However, the analytical insight shown in Eq. 5 (i.e., bias expected to be $h^{2}\cdot \sum \beta_{k}^{2}\delta_{k}/\sum \beta_{k}^{2}p_{k}\left(1-p_{k}\right)$ ) suggests that the issue discussed here remains relevant in the case of multiple causal SNPs as $\sum \beta_{k}^{2}\delta_{k}$ is unlikely to be zero, even while allowing the signs of *δ*_*k*_ to differ.

Assuming the true heritability *h* ^2^ is known, we also demonstrated the potential type I error issue of the LMM in the presence of HWD using data that consist of related individuals only. In practice, this issue diminishes if the sample includes a large number of independent individuals or the magnitude of HWD at the causal SNP is small. Additionally, in practice, *h* ^2^ is treated as unknown, in which case, the type I error rate of the LMM is well controlled; indeed, no increased false positives of the LMM due to HWD have been reported in the literature to the best of our knowledge. However, the estimate of *h* ^2^ can be upward biased and upwardly so if *δ*_causal_ > 0, as demonstrated in the simulation study in Section 3.2 and seen in the cystic fibrosis application study in Section 3.1. This new observation offers a possible complementary explanation of the “missing heritability” discussed extensively in Maher (2008).

In practice, SNPs out of HWE are typically not analyzed due to concerns for low genotyping quality (Wellcome Trust Case Control Consortium, 2007; Bycroft et al., 2018; Marees et al., 2018). However, the observation made here remains relevant as the heritability estimates in LMM-based models are biased when the causal SNPs are in HWD (which is unknown in practice) but not the tested SNPs. This is also supported by Figure 3B. When there was HWD at the causal SNP (e.g., *δ*_causal_ = 0.10 on the X-axis), there was a type I error issue even if there was no HWD at the tested SNP (i.e., *δ*_tested_ = 0). Conversely, Figure 3A shows that if there was no HWD at the causal SNP (i.e., *δ*_causal_ = 0 on the X-axis), then the test is accurate even if there was HWD at the tested SNP (*δ*_tested_ = 0.06).

Additionally, the HWE-based screening practice itself can be called into question because a truly associated SNP is often in HWD (Wittke-Thompson et al., 2005; Ryckman and Williams, 2008; Turner et al., 2011). The potential of leveraging the HWD expected at a causal SNP to increase the power of association testing has been explored by several groups (Song and Elston, 2006; Wang and Shete, 2008; Zhang and Sun, 2020).

We have not examined the implication of HWD combined with linkage disequilibrium (LD) (Weir, 2008) on the LMM, which is an important future research question. Additionally, recent work has shown that dominant genetic effect could complicate the LD measure and interpretation (Palmer et al., 2021), which in turn could affect our examination of the effect of HWD on the LMM.

Although the linear mixed-effect model is a popular and powerful method for GWAS, conceptually, the use of kinship coefficient matrix (i.e., Σ_Φ_), derived from *G*, as part of the variance–covariance matrix (i.e., Σ_*y*_) of the LMM can be problematic because the response variable *Y* is the phenotype of interest. An alternative approach is to reverse the roles of *Y* and *G* in the regression model. Indeed, O’Reilly et al. (2012) proposed MultiPhen, a method that treats the genotype *G* of an SNP as the response variable and phenotype values *Y* of multiple traits as predictors, and uses an ordinal logistic regression applicable to independent samples. More recently, Zhang (2021) (Chapter 2) proposed a generalized reverse (or retrospective) regression model that can be applied to dependent samples, which takes the form of

$$g=\alpha 1+\beta \text{y}+\gamma \text{z}+\epsilon ,\;\epsilon ∼N\left(\text{0},\sigma^{2}\Sigma_{g}\right),\;\sigma^{2}\Sigma_{g}=\sigma^{2}\Sigma_{\Phi }+\Sigma_{\delta },$$ (6)

where Σ_Φ_ is the kinship coefficient matrix as defined earlier and Σ_*δ*_ is a function of *δ* that explicitly models the amount of HWD; the use of a linear model for the discrete genotype data *G* is motivated by the work of Chen (1983).

Interestingly, if the variance and covariance matrices in Eqs 4, 6 of the LMM were the same, the resulting score test statistics are also the same. However, conceptually, the model Eq. 6 correctly uses the kinship coefficient matrix to model the response variable *G*, in contrast to the LMM model of Eq. 4. Specifically, at a tested SNP, as the reverse regression is conditional on *Y*, the variance–covariance matrix only concerns *G*_tested_, that is, Σ_*g*_. The modeling and estimation of Σ_*g*_ can account for potential HWD through Σ_*δ*_, in addition to the genetic correlation captured by the kinship coefficient matrix of Σ_Φ_, resulting in a more robust association test for related individuals. Indeed, when the method was applied to the same simulated sib-pair data in Section 3.3, it had correct type I error control [results shown in Figure 2.2 of Chapter 2 of Zhang (2021)]. However, how to model gene–environment interaction through the reverse regression framework remains an open question.

## Data Availability

The data analyzed in this study are subject to the following licenses/restrictions: The CF application data are available by application to the Cystic Fibrosis Canada National data registry for researchers who meet the criteria for access to confidential clinical data for the purpose of CF research. Requests to access these datasets should be directed to cfregistry@cysticfibrosis.ca.
